# Small intra-tropical long-distance migratory birds track rainy seasons across hemispheres

**DOI:** 10.1098/rspb.2024.2633

**Published:** 2025-01-22

**Authors:** Yann Rime, Samuel Temidayo Osinubi, Felix Liechti, Barbara Helm, Raphaël Nussbaumer

**Affiliations:** ^1^ Swiss Ornithological Institute, Sempach, Switzerland; ^2^ Department of Environmental Sciences Zoology, University of Basel, Basel, Switzerland; ^3^ FitzPatrick Institute of African Ornithology, University of Cape Town, Cape Town, South Africa; ^4^ Cornell Lab of Ornithology, Ithaca, New York, USA; ^5^ A Rocha Kenya, Watamu, Kenya

**Keywords:** niche tracking, rainfall seasonality, ecological barriers, *Halcyon senegalensis*, *GeoPressureR*

## Abstract

The main features of long-distance migration are derived from landbirds breeding in the Northern Hemisphere. Little is known about migration within the tropics, presumably because tropical species typically move opportunistically and over shorter distances. However, such generalizations are weakened by a lack of solid data on spatial, temporal and behavioural patterns of intra-tropical migrations. To start filling the research gap, we provide comprehensive data for small-sized intra-African migrants, woodland kingfishers. We inferred stationary locations, migration timing, flight behaviour and wind experienced en route from multi-sensor loggers recording atmospheric pressure, light and acceleration. After breeding in South Africa, all tagged individuals migrated 4000 km to South Sudan, spending their non-breeding period within 100 km of each other. Thereby, woodland kingfishers tracked their climatic niche, using two rainy seasons in open woodland across the Equator. Migratory flights were strictly nocturnal, reaching 2890 m.a.s.l. Flights were unusually short, but lengthened when crossing rainforests, a behavioural adjustment similar to barrier-crossing along well-described flyways. These results suggest that long-distance intra-tropical migration displays patterns that are surprisingly similar to other flyways. Pending confirmation in other species, intra-tropical migrations might be more extensive and less flexible than assumed, underlining the importance of further research guiding conservation efforts.

## 1. Introduction

Migration, consisting of periodic movements between two or more locations [1], is usually defined from a high-latitude perspective, where it aligns with photoperiod-dominated environmental seasonality [2]. However, comparative data from other regions are scarce, while the vulnerability of migratory species calls for a global understanding of migratory systems. In particular, intra-tropical migration remains very poorly studied compared to the movement ecology of birds breeding in the Northern Hemisphere [3–5]. It has been argued that in subtropical and tropical regions, where photoperiodic seasonality is limited, migration is less common, less extensive and more flexible [2,3,6]. However, in view of lacking data, intra-tropical migrations are probably underestimated. We aimed to fill this research gap by focusing on migration within Africa.

At low latitudes, the annual cycle of locally breeding birds and their intra-tropical migration patterns largely depend on rainfall seasonality [7–10]. The climate of sub-Saharan Africa is mostly driven by the fluctuation of the inter-tropical convergence zone, with tropical areas north of the Equator receiving rainfall during the boreal summer and tropical to subtropical areas south of the Equator receiving rainfall mostly during the austral summer [11,12]. Driven by resource abundance, many African bird species time their breeding to coincide with or precede the rainfalls [8]. Other annual cycle activities, in particular moult and migration, are poorly known. Migratory behaviour within Africa and its drivers are poorly understood, and for many species, it is not even clear whether they migrate at all. The available observations, ring recoveries and recent tracking of larger species suggest that intra-African seasonal movements often span short distances of less than 1000 km [13–15]. However, several species perform long-distance, trans-equatorial migration. Abdim’s storks *Ciconia abdimii* breed in the Sahel and migrate to central, eastern and southern Africa, hereby benefitting from the rainy seasons of both hemispheres [16]. Some small-bodied austral migrants, such as the pennant-winged nightjar *Caprimulgus vexillarius* [17]*,* also show distinct breeding and non-breeding areas involving long-distance migration across the Equator, but no tracking data exist.

In contrast to intra-African migrants, the migration behaviour of Palaearctic-breeding species within Africa has been more intensively studied [18–20]. In some Palaearctic-breeding migrants, intra-African seasonal movements have been described, whereby these species track seasonal rainfall and vegetation greenness [20]. Yet, other Palaearctic-breeding birds use single non-breeding locations (e.g. [21]). Some migratory species have population-specific non-breeding grounds, that is, showing high migratory connectivity [22], whereas others spread over large areas that are not population-specific, that is, showing low migratory connectivity [23,24]. Small-bodied long-distance migrants generally travel at night, typically covering long distances between stopover sites where they rest and re-fuel [2]. The journey between breeding and non-breeding sites often crosses habitats that are suboptimal or hostile for a species. So-called migratory barriers are typically abiotic geographical features, such as seas, mountains and deserts [25–28]. Barriers can also arise from biotic features that are unfavourable to some species, amounting to ‘green’ or ‘soft’ barriers [2,27–29]. For example, species that prefer open habitats could use rainforests for stopover if necessary, but tend to avoid them. Diverse behavioural adjustments of migratory flights and stopover behaviour allow migratory birds to cover sometimes large distances above such unhospitable habitats [21,30–32]. Such detailed behavioural data can only be obtained by tracking the movements of individual birds. They are eminently important not only for building fundamental knowledge on migration, but also for guiding conservation efforts. For example, information on connectivity, and on the locations where populations spend the non-breeding season, are essential for year-round conservation [33,34].

For intra-African migrants, this information is almost completely lacking, partly because of technical constraints. The little tracking data we have are confined to a few large species that can carry heavy transmitters [13,35], whereas the majority of the avian diversity is small-bodied [36]. Thus, the links between breeding, movements and seasonality of small intra-African migrants have not been studied with individual tracking but were mostly described through ring recoveries and field observations [8,37]. Light-level geolocation, which is commonly used for tracking small Palaearctic-breeding migrants, typically produces poor positioning in the tropics, where variations in day-lengths across latitudinal gradients are limited [38,39]. The development of a more precise geolocation method for light-weight loggers based on atmospheric pressure has opened new opportunities to study movements of small birds, in particular, for short distance migration and migration across the Equator or during the equinox [40–42]. Combined with an accelerometer sensor, this method provides detailed information on flight and stopover behaviour, which have never been described before in small intra-tropical migrants [21,43]. Here, we used these multi-sensor geolocators for the first time to our knowledge in an intra-tropical migrant, the woodland kingfisher *Halcyon senegalensis cyanoleuca*, to establish a case study for comparisons with Palaearctic-breeding migrants. We also aimed to introduce a reproducible framework for investigating migratory behaviour, spatio-temporal movement patterns and their putative environmental drivers within intra-tropical migratory systems.

Woodland kingfishers are small-sized inhabitants of open wooded habitats that breed in South Africa during the rainy season, and thereafter depart for migration [44,45]. Woodland kingfishers are expected to move to areas that offer fresh vegetation when the dry season begins at their breeding sites, but approximate non-breeding movements were known only for two birds tracked with light-level geolocators [46].

From multi-sensor geolocator data, we derived a broad range of descriptors of migratory behaviour of woodland kingfishers to facilitate comparisons with Palaearctic-breeding migrants. In addition to defining the general patterns of long-distance migration, we used atmospheric pressure data on a 30 min resolution to unveil flight behaviour in three spatial dimensions including the vertical space use, and investigated stopover strategy [21,42]. To determine environmental correlates of these movements, we investigated local habitat and average seasonal climatic conditions encountered throughout the annual cycle, especially rainfall patterns and vegetation greening, as well as landscape-scale habitat composition.

Based on current conceptualizations of landbird migration [2,3], we aimed to distinguish between two broad scenarios that could characterize woodland kingfisher movements, containing the following elements:

- scenario 1: flexible migration, as considered typical for intra-tropical migrants. Woodland kingfishers would move short distances to spend the non-breeding season closer to the Equator [2,3], with high individual variation in terms of time and space. To assess local conditions, birds might be diurnal migrants, potentially carrying out relatively short flights at relatively low altitudes. No crossing of (green) barriers is expected; and
- scenario 2: tightly regulated long-distance migration, as considered typical for Afro-Palaearctic migrants. Woodland kingfishers would cross the Equator to commute between distinct areas within short time-windows that are similar across years. Birds might migrate at night, potentially carrying out relatively long flights at high altitudes. We predicted stopovers to be extended in suitable habitats and flights to be higher and longer over unsuitable habitats. Specifically, we expected the birds to alter their flight behaviour if crossing the dense Congo Basin rainforest [27,29]. By long-distance migration, woodland kingfishers would track, or anticipate, favourable conditions in the target areas.

However, as these hypotheses derive from Palaearctic-breeding systems, some elements might deviate at low latitudes. For example, because of the symmetry of environmental conditions relative to the equator, a longer migration distance (scenario 2) enables greater niche-tracking, which does not apply to northern-hemisphere, high-latitude breeders [47]. Under scenario 1, woodland kingfishers would reach regions where rainfalls are more stable throughout the year, but encounter habitats that differ from the breeding site. Conversely, under scenario 2, trans-hemispherically migrating woodland kingfishers would encounter seasonal conditions and habitats that are more similar to those at the breeding grounds. Likewise, because the woodland kingfisher’s migration crosses habitable land, several behaviours might differ from Afro-Palaearctic species. These include connectivity, which to our knowledge has not been reported in intra-African migrants [14,15], lengths of flight and stopovers and use of supportive winds, which are well-described for northern-hemisphere breeding migrants [2,48], but are completely unknown for intra-tropical migration. We thus hypothesize that the above scenarios do not fully capture patterns of intra-tropical migration and that some elements might reassemble.

## 2. Methods

### (a) Study species

Woodland kingfishers use a wide range of open wooded habitats, from savannah to forest clearings and gardens, with a preference for riverine woodland. These small-sized birds (23 cm, 60−81 g) prey on large arthropods and small vertebrates. Three subspecies are described: *H. s. cyanoleuca* from southern Africa and *Halcyon senegalensis senegalensis* from the Sahel and western Africa have migratory and resident populations, while *Halcyon senegalensis fuscopileus* from central to western Africa is considered resident [44].

### (b) Geolocators and deployment

Tagging was carried out during three breeding seasons between December 2016 and January 2019 at Mogalakwena Research Centre, South Africa (22°43'35" S, 28°46'30" E). Twenty adult individuals were equipped with 27 multi-sensor geolocators (GDL−3 PAM with light stalk, Swiss Ornithological Institute, Switzerland, weight 1.6 g, recording atmospheric pressure and acceleration at 30 min intervals and light intensity at 5 min intervals) mounted on a pre-sized leg-loop elastic harness [49]. Six individuals were equipped twice. One individual was equipped three times during two breeding seasons but lost all tags. Five geolocators were successfully retrieved from four individuals (table 1). The geolocators 16LN and 22NO were fitted on the same bird in 2017 and 2019, respectively. The male 16LO and female 16LP were a breeding pair. All other birds from which the tags were retrieved were females. The overall recapture rate of 0.26 is comparable to other studies on small birds [50], especially considering that some birds were observed but had learned to avoid traps. Nineteen individuals were marked with colour rings but not with geolocators between 2015 and 2020. However, these birds were not targeted for recapture and the behaviour of the species, perching high with legs in the feathers, did not allow a clear comparative assessment of return rates through visual resighting.

**Table 1.** Summary of the general information on migration schedule, flight performance and stationary periods of woodland kingfishers tagged with multi-sensor geolocators during an entire annual cycle, divided between post-breeding (*n* = 5) and pre-breeding migrations (*n* = 4). Wind support is the mean tailwind component weighted relative to flight duration. Male 16LO and female 16LP were a pair; loggers 16LN and 22NO recorded repeated tracks of the same individual in two years.

	**tag ID**	16LN	16LO	16LP	20IK	22NO
post-breeding	departure breeding site	05.04.2017	26.03.2017	15.04.2017	22.03.2018	17.04.2019
	arrival non-breeding site	07.06.2017	01.06.2017	15.06.2017	21.06.2018	08.07.2019
	migration duration (days)	63.72	67.69	61.70	90.95	81.89
	number of flights	26	26	22	25	38
	days in the Congo Basin	3.82	2.75	1.64	4.76	15.78
	cumulative flight distance	4228	3914	4057	3998	4220
	cumulative flight hours	83.00	85.83	83.75	91.42	88.58
	median flight altitude (m.a.s.l.)	927	1179	862	909	810
	median flight altitude (m.a.g.l.)	204	157	183	146	129
	max flight altitude (m.a.s.l.)	2455	2714	2352	2587	2890
	max flight altitude (m.a.g.l.)	1670	2151	1732	1159	1661
	wind support (km h^−1^)	8.72	7.98	8.34	7.10	5.01
	median stopover duration	0.90	0.92	0.89	1.00	1.03
pre-breeding	departure non-breeding site	27.10.2017	17.10.2017	27.10.2017	26.10.2018	—
	arrival breeding site	17.11.2017	14.11.2017	13.11.2017	04.12.2018	—
	migration duration (days)	21.57	28.65	17.56	39.90	—
	number of flights	17	24	14	26	—
	days in the Congo Basin	2.67	0.54	0.55	1.59	—
	cumulative flight distance	4129	4655	4038	4309	—
	cumulative flight hours	90.17	102.75	82.50	99.08	—
	median flight altitude (m.a.s.l.)	1354	1181	1354	972	—
	median flight altitude (m.a.g.l.)	506	213	346	142	—
	max flight altitude (m.a.s.l.)	2688	2459	2677	2352	—
	max flight altitude (m.a.g.l.)	2002	1545	2021	1688	—
	wind support (km h^−1^)	6.10	6.37	6.43	4.93	—
	median stopover duration	0.77	0.91	0.71	0.88	—

### (c) Geolocator analyses

We modelled trajectories from pressure, light and wind data following Nussbaumer *et al*. [41,51], using the associated R package *GeoPressureR* [40]. We followed the recommended procedure to reconstruct trajectories provided in the GeoPressureManual [52], and we briefly describe the main steps. First, we identified migratory flight periods based on the combination of accelerometer (i.e. high activity values for at least 1 h) and pressure data (sharp change in pressure indicating change of altitude). Birds were considered to be at a single stationary location (< 20 km) between migratory flights. Second, we constructed pressure likelihood maps by matching geolocator pressure measurements of each stationary period with data from the ERA5-LAND reanalysis [40,53]. Third, we built light likelihood maps using the threshold method and a calibration from the equipment and retrieval periods [54]. The likelihood maps of all twilights from the same stationary periods were aggregated using a log-linear pooling function [40]. Finally, we modelled individual trajectories with a hidden Markov model using pressure and light likelihood maps as well as wind data [41]. Based on this model, we produced a probability map for the position at each stationary period, the most likely individual trajectory for each bird and 100 random simulations of the trajectories. The dataset resulting from these analyses was formatted following Nussbaumer [55] and is available at https://doi.org/10.5281/zenodo.13829929 [56].

### (d) Remote-sensing data

Daily temperature and precipitation data for the breeding and non-breeding sites were obtained from the ERA5-Land between 1979 and 2020 [53]. We then calculated the average precipitations and temperatures for each day of the year. Normalized difference vegetation index (NDVI) values were retrieved from the MODIS Terra Vegetation [57] available for the 2000−2024 period at a resolution of 16 day temporally, and 1 km^2^ spatially. We computed the seasonal average greenness along the north–south trajectory of the bird by computing the average NDVI over all years, the longitudinal range covered by all the birds (24°−30° as defined by the minimum and maximum longitude values of the track) and on a 1° latitude resolution. To describe landscape-scale habitat at stationary sites (stopover, breeding and non-breeding sites), we extracted landcover proportion on a 20 km radius around each most likely location from the 2019 Copernicus Global Land Cover Layers (100 m resolution [58]).

### (e) Description of flight behaviour and stopover use

For each migratory flight, we calculated flight duration as well as maximum, median and mean flight altitude above ground and above sea level (computed with the function pressurepath_create()). From the movement model, we retrieved flight distance, ground speed and wind support (tailwind component) for each flight [40]. We then classified the migratory flights and stopovers into two habitat-based categories: Congo Basin equatorial forests between −3° and +3° latitude and wooded savannah otherwise.

## 3. Results

### (a) Migration timing and routes

**Figure 1.**
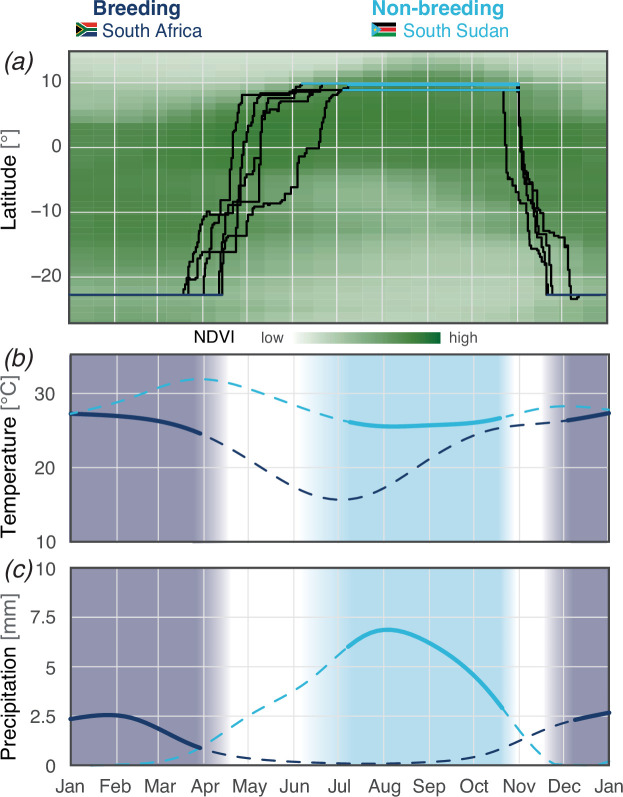
Presence of woodland kingfishers at the breeding (dark blue) and non-breeding (light blue) sites relative to the respective long-term patterns of (*a*) vegetation greenness, (*b*) temperature, and (*c*) rainfall. (*a*) Latitudinal position of all birds during their entire annual cycle with the multi-year average normalized difference vegetation index (NDVI) in the background across the latitudinal gradient covered during the entire annual cycle, including migratory stopovers. Birds align the timing of their migration with the seasonal greening in each hemisphere. When crossing the equatorial regions where vegetation greening is more constant year-round, migration tends to be faster (shorter stopovers and longer flights, visible by the steepness of the black line). (*b*) Average temperature at the breeding (dark blue) and non-breeding (light blue) sites. Solid lines indicate the period when the bird is present, suggesting the use of a similar thermal niche in both breeding and non-breeding seasons and avoidance of extreme temperatures (both low and high). Migration phases are indicated by blank background. (*c*) Average precipitation at the breeding (dark blue) and non-breeding (light blue) sites. While the absolute precipitation levels differ, the birds stayed at the respective sites during the relative local peaks of rainfall.

**Figure 2.**
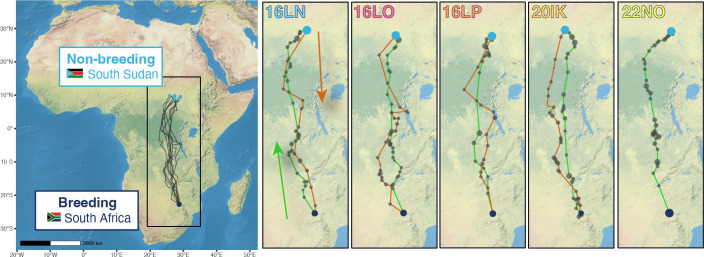
Most likely trajectories of the four woodland kingfishers equipped with multi-sensor geolocators in South Africa. Large dark blue dots denote the breeding site where the birds were equipped in South Africa, light blue dots show non-breeding sites and smaller dark grey dots are stopover sites with sizes proportional to their duration. The green lines symbolize the post-breeding migration northwards, while the brown is for the southward pre-breeding migration, as indicated by the arrows on the left-hand individual plot. The tracks 16LN and 22NO are from the same individual in different years.

The tracked woodland kingfishers left the South African breeding site between 22 March and 17 April (figure 1*a*). This timing aligned with the average end of the rainy season (figure 1*c*) when the average temperatures fell below 25°C (figure 1*b*). The north-bound post-breeding migration covered on average 4080 km over 73 days (figure 2; electronic supplementary material, figure S1), for a total of 86.5 flight hours with minor individual variation (table 1). All five tracks showed long stopovers in Zambia, where vegetation greenness remained higher at the end of the rainy season compared to the breeding site, as shown by the values along the latitudinal gradient covered during migration in figure 1*a*. Migration above the equatorial rainforest was faster, with only short stopovers (figures 1*a* and 2). Four of the five tracks showed stopovers for a few weeks (25 days on average) about 200 km south of their final non-breeding sites in South Sudan. The timing of this stopover coincided with locally advanced greenness while the final non-breeding sites still had comparatively low NDVI (figure 1*a*). In 2019, one individual (22NO) migrated through the equatorial region a month later and directly reached the final non-breeding site without preliminary residency at another site. Three out of four birds initiated pre-breeding migration almost on the same date. The departure from the non-breeding sites corresponds to the transition between the rainy season and the dry season, coupled with a decrease in vegetation greenness. Compared to post-breeding migration, pre-breeding migration was much shorter (mean = 26.9 days) with slightly more time in flight (93.6 h; figure 1*a*; table 1). Three birds arrived at the breeding site mid-November, among them the breeding pair (16LP, 16LO) within two days, and one in early December, just before the onset of the rainy season and vegetation greening.

### (b) Non-breeding site

**Figure 3.**
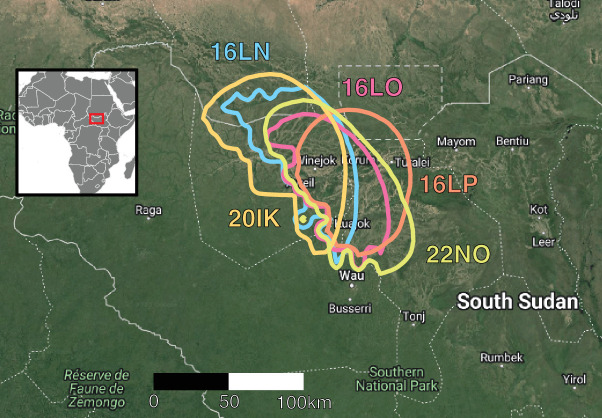
Location of non-breeding sites in South Sudan. This area is located at the edge of the Sahel zone, with similar seasonal weather patterns and habitat as the breeding region. The contour lines correspond to the 90th quantile of the marginal map extracted from the movement model. Male 16LO and female 16LP were a pair, and loggers 16LN and 22NO were equipped on the same bird in two years.

The birds tracked within three years spent the non-breeding seasons in the same area of South Sudan, within less than 100 km distance of each other (figure 3). Birds migrated individually to this area, even in the case of the breeding pair (16LP and 16LO). Landscape-scale habitat structure (20 km radius, electronic supplementary material, figure S3) at the breeding site (median tree cover = 17.9 %) was similar to that at the non-breeding sites (median tree cover = 17.3 %). Tree cover was denser, but more variable, at migratory stopover sites (median = 41.1 %). Thermal niche was similar between the breeding site and non-breeding sites (between 26°C and 28°C), and birds were present during the peak rainy season of both sites (figure 1*b*,*c*).

### (c) Flight behaviour

**Figure 4.**
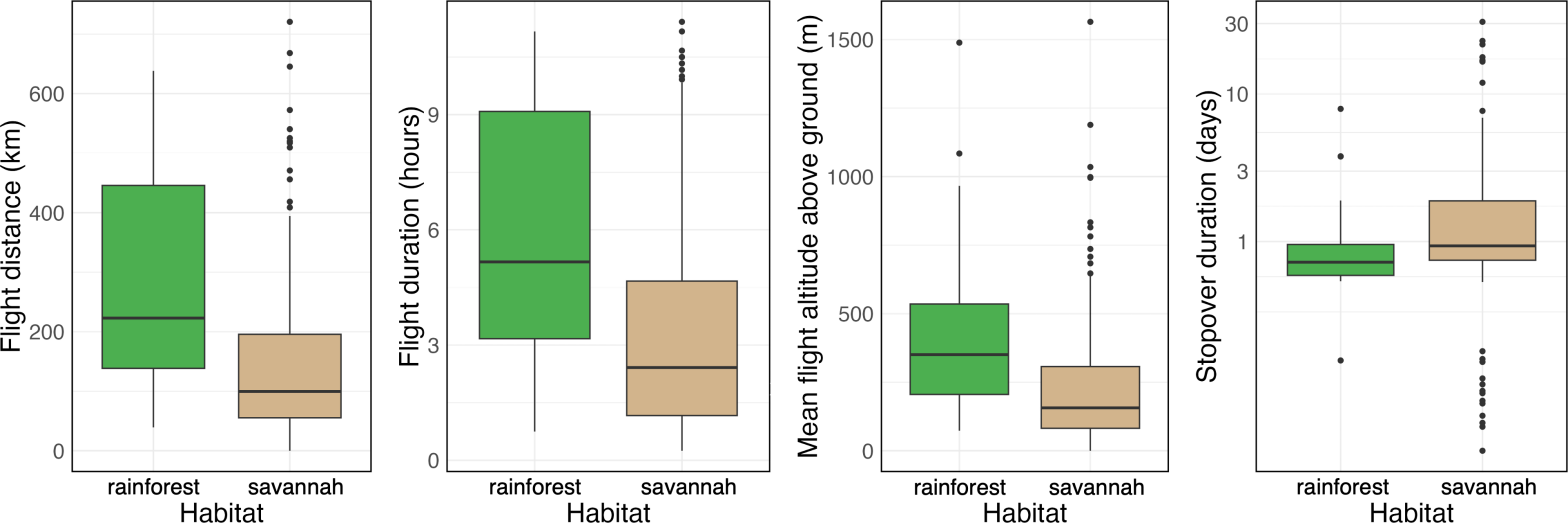
Comparison of the flight performance (distance, duration and mean flight altitude above ground) and stopover duration during the crossing of the Congo Basin equatorial rainforest (33 flights) and crossing of other areas, considered as savannah and open woodland habitat (185 flights). The longer and higher flights combined with shorter stopovers when crossing the equatorial rainforest suggest that woodland kingfishers avoided the forests, a behaviour suggesting barrier crossing adjustment.

Migratory flights occurred exclusively at night. Departure time was variable, with a mean at 22.38 ± 02.39 (UTC+2), that is, more than 4 h after sunset (electronic supplementary material, figure S2). The mean landing time was 02.20 ± 02.09, so that no flight was prolongated into the day. Woodland kingfishers migrated with frequent (median of 25 flights per migration) but short flights (median = 2.7 hours). They nonetheless performed a few long flights, with maximal flight duration per individual between 10.5 and 11.4 h (electronic supplementary material, figure S2). Longer flights were initiated earlier during the night. Flight duration and distance were generally shorter in the post-breeding migration but with more wind support than in the pre-breeding migration (table 1). Flight altitude varied between 417 and 2890 m.a.s.l. (0 to 2151 m above ground level; table 1; electronic supplementary material, figure S4). While crossing the Congo Basin, birds shortened the duration of stopovers (median = 0.7 days; figure 4) compared to savannah areas (median = 0.9 days; figure 4). Flights were also much longer above the Congo Basin (median = 5.2 h, *n* = 33) compared to savannah habitats (median = 2.4 h, *n* = 185; figure 4), and at higher altitude above the ground (figure 4; electronic supplementary material, figure S4). Tree cover was higher at stopover sites in the Congo Basin (median = 98.7 %), compared to other stopover sites (median = 35.5 %).

## 4. Discussion

Woodland kingfishers breeding during the austral rainy season in South Africa performed over 4000 km of migration to spend the non-breeding season less than 100 km apart from each other in South Sudan. There, they experienced a position of the inter-tropical convergence zone that allowed them to benefit from local rainfalls in habitats that were similar to the breeding site. They thereby maintained an exceptionally high seasonal niche constancy compared to Palaearctic migrants. Woodland kingfishers also showed clear temporal precision of movements, contrasting with the expectation of intra-tropical movements being more flexible and opportunistic than long-distance migratory systems in the northern hemisphere. Moreover, the nocturnally migrating tracked birds adjusted their behaviour during migration through the Congo Basin rainforests, compared to wooded savannah, in a similar way as barrier-crossing in Afro-Palaearctic migrants. Thus, overall, our findings majorly supported scenario 2, which proposed tightly regulated long-distance migration, similar to that of Afro-Palaearctic long-distance migrants.

Route patterns clearly confirmed scenario 2. All tracked birds crossed the Equator to reach savannah habitats in the Northern Hemisphere. According to overview assessments, the main difference of intra-tropical migration compared to northern-latitude migration is the scarcity of cross-equatorial long-distance movements [2,3]. Woodland kingfishers travelled surprisingly far: for individuals breeding during the southern African rainy season, less distant equatorial and subequatorial regions could provide suitable non-breeding locations [2]. During post-breeding migration, woodland kingfishers in our study had prolonged stopovers in Zambia, where vegetation remained on average greener than at the breeding site (figure 1). However, rather than staying in this area for their entire non-breeding period, the tracked woodland kingfishers migrated twice the distance already travelled, across the Equator to a specific area in South Sudan. This behaviour allows them to benefit from conditions that are more suitable compared to the stopover sites, with a higher food abundance in South Sudan during the Northern Hemisphere summer rainy season compared to the increasingly dry conditions in Zambia. Moreover, habitats in South Sudan are more similar to those at the breeding site. Given the lack of comparative intra-African data, we cannot determine whether woodland kingfishers are exceptional, or whether such long journeys are more widespread than often assumed.

Remarkably, the non-breeding destinations were highly concentrated. Within and between years, woodland kingfishers spent the non-breeding season close to each other, in contrast, for example, to cuckoo species in West Africa [14,15] and to general views of relatively variable spatio-temporal patterns in intra-tropical or austral migrants [3,6]. However, in our woodland kingfishers, studying more populations would be required to assess connectivity strength as a general pattern. Connectivity has mostly been studied in the context of land birds from the Northern Hemisphere, where both high and low connectivity strategies co-exist depending on the species [22–24,59]. Again, the general lack of individual tracking studies in intra-African migrants does not allow comparisons, and it is unclear if the high concentration of non-breeding sites we found in our population of woodland kingfishers is an exception or more common.

As expected for maintenance of their climatic niche, migration of the woodland kingfishers was closely linked to the seasonality in rainfall and vegetation greening at breeding and non-breeding sites. All individuals moved within a relatively small time-window, reaching the target areas under favourable habitat conditions, that is, with green vegetation and during the rainy season. Woodland kingfishers stayed in South Sudan until the end of the rainy season and migrated back to southern Africa. Post-breeding migration of woodland kingfishers was notably longer than pre-breeding migration, a common pattern in other migration systems [60,61]. Movements also seemed adjusted to seasonality along the route: the first prolonged stopover after crossing the rainforest occurred about 200 km south of the final non-breeding sites. Such fine-tuning of migratory movements to the advance of vegetation greenness has also been reported in several Palaearctic migrants [62,63]. Nevertheless, more data would be required to link potential minor variations in timing with local conditions. The concentrated migration timing of woodland kingfishers aligned with the long-term onset of the rainy season, which moves into the northern savannah from the Equator at the beginning of the boreal summer, accompanied by increases in vegetation greenness and food abundance [11,64]. Our data did not allow us to distinguish whether the birds followed or anticipated environmental conditions. We could also not identify to what extent the birds used environmental or photoperiodic cues [65]. With more data from intra-African migrants, such intriguing questions could be addressed in the future.

The non-breeding range of the austral woodland kingfishers *H. s. cyanoleuca* lies within the breeding range of the closely related subspecies *H. s. senegalensis*, which breeds during the non-breeding presence of the austral migrants [45]. This overlap might be made possible by a high temporary food abundance, reducing intra-specific competition [2], or by subtle niche partitioning between these two subspecies that reportedly share similar habitats and breeding cavities [44,45]. The woodland kingfisher migration strategy compares to systems on other continents, for example, in the Neotropics. Among austral-breeding migrants, fork-tailed flycatchers *Tyrannus savanna* from South America use open landscapes in both hemispheres and overlap with locally breeding populations [66,67]. Such range overlap implies limitations in the observational understanding of intra-tropical migration phenology (e.g. [68]), highlighting the advantage of individual tracking to disentangle the multiple origins of observed birds.

In our study, for the first time to our knowledge, we report detailed data on flight behaviour in an intra-tropical migrant. During the pre-breeding journey of less than a month, most of the nights were spent flying. The arrival at breeding sites at the beginning of the rainy season and thus at the onset of vegetation greenness, was later than in other migrants such as pennant-winged nightjars that start breeding before the first rains [17]. The rapid pre-breeding migration of woodland kingfishers could have benefitted from favourable conditions at the non-breeding site, at the end of the Sahel rainy season [69,70]. While woodland kingfishers breeding in southern Africa are still present in South Sudan, many Palaearctic migrants on the eastern flyway use this area in the boreal autumn for prolonged stationary periods before continuing their migration to southeastern Africa [20,71–73].

In addition to general spatio-temporal patterns, our analyses highlight behaviours that are specific to intra-African flyways. Flights above the Congo Basin equatorial forests were longer and higher than over savannah, and stopovers there usually lasted only one day. Longer and higher flights are a common strategy in birds crossing barriers such as sea or desert [21,32]. Barrier-crossing flights also occur in closely related species: sacred kingfishers *Todiramphus sanctus* from Australia undertake sea-crossing flights at least as long as woodland kingfishers above the Congo Basin [44,74]. The equatorial forests may thus act as a barrier for woodland kingfishers. A similar avoidance of a ‘green’ barrier has been described in European nightjars *Caprimulgus europaeus* and Eurasian hobbies *Falco subbuteo* avoiding the Congo Basin [27,28]. In woodland kingfishers, flight altitude varied, reaching moderately high elevations up to 2890 m.a.s.l. and flights were mostly wind-supported. In comparison, maximal altitudes of Afro-Palaearctic migratory songbirds can exceed 5000 m over migratory barriers [75,76], and non-passerines can reach altitudes above 8000 m [77]. Our data also provide a first confirmation for wind-supported nocturnal migration in an intra-tropical migrant, a flight strategy which is commonly realized by migrants across the Palaearctic and Nearctic [2,48]. Flights of woodland kingfishers occurred exclusively at night, but in detail, their diel activity patterns differed from those of palearctic migrants [21,78,79]. Woodland kingfishers departed later in the evening and did not prolong flights into the day. Conversely, species from the Northern Hemisphere often prolong flights especially above migratory barriers, such as seas and deserts [30,75,78,80]. Overall, the migration strategies of woodland kingfishers displayed several typical features of other, well-studied long-distance migratory systems of birds breeding in the Northern Hemisphere, with some system-specific specializations. The precision of the spatial and temporal patterns of migration correspond to those of long-distance Afro-Palaearctic migrants, with departure and arrival dates at both the breeding and non-breeding-sites being very consistent among the tracked individuals. Nevertheless, the high level of seasonal niche tracking, with almost identical habitat and climatic niche in the breeding and non-breeding season, contrasts with birds breeding in the Northern Hemisphere, that predominantly use climatic niches and commonly also habitats that differ from their breeding site [2]. A seasonal overlap with other subspecies exists in many migrants, but the overlap of two woodland kingfisher subspecies resulting in inversed breeding time is uncommon [2]. Inverted breeding seasons have recently arisen in swallow species that apparently changed their migratory behaviour to breed on the non-breeding site [81]. In woodland kingfishers, further research on niche partitioning between the subspecies would be needed to understand the ecological implications of seasonal overlap. Migratory flights of woodland kingfishers were especially short and more strictly nocturnal, but the adjustments of flight altitude, flight duration and stopover duration to barrier crossing were similar to those observed in some Afro-Palaearctic species.

Our study constitutes, to our knowledge, the first evidence of trans-equatorial long-distance migration tracking the rainy seasons in both hemispheres in a small migratory organism. Using specific habitats and local climatic conditions at two locations across the Equator might lead to higher sensitivity in case of climate and land use changes in the breeding or in the non-breeding areas. At present, these and other possible threats to intra-tropical migrants are difficult to predict. While long-distance migrants decline in the Northern Hemisphere [34], fundamental knowledge on intra-tropical migrants and their status is still lacking, hampering to address their conservation. Deforestation in the Congo Basin could, for example, create open habitats that may be suitable as stopover or non-breeding sites for woodland kingfishers and other birds of open landscapes (e.g. [82]), whereas changes in seasonal weather patterns might disrupt these migratory systems, as for many long-distance migrants from the Northern Hemisphere [11,34]. Region-specific changes, with for example droughts in southern Africa combined with more intense rainfall over the Sahel, might alter the finely tuned annual cycle of these birds. Given the rapid changes in climate and land use throughout sub-Saharan Africa, the critical lack of data informing on the constraints and opportunities faced by intra-African migrants calls for further research using powerful tracking technology, such as multi-sensor loggers. Given the advantages of geolocation using atmospheric pressure, especially in tropical regions with limited variations in day length across the year, we recommend using the framework proposed here for future studies on small intra-tropical migratory birds [40–42]. To envision the effects of habitat and climate changes on tropical bird communities, studies on the movement ecology of intra-tropical migrants should be a priority in global bird migration research.

## Data Availability

The datasets, supplementary material and code supporting this article is are available at [83]. The Geolocator Data Package is available at [56]. Supplementary material is available online [84].
